# Modification of the Forms of Self-Determined Regulation and Quality of Life after a Cardiac Rehabilitation Programme: Tennis-Based vs. Bicycle Ergometer-Based

**DOI:** 10.3390/ijerph18179207

**Published:** 2021-08-31

**Authors:** Juan Pedro Fuentes-García, Lorena Alonso-Rivas, José Javier Gómez-Barrado, Víctor Manuel Abello-Giraldo, Ruth Jiménez-Castuera, César Díaz-Casasola

**Affiliations:** 1 Faculty of Sport Science, University of Extremadura, Avda. Universidad S/N, 10003 Cáceres, Spain; ruthji@unex.es; 2 General Health Psychilogist, Avda. Av. María Auxiliadora 1, 10002 Cáceres, Spain; rilonso@gmail.com; 3 Cardiology Department, San Pedro de Alcántara Hospital, Avda. Pablo Naranjo S/N, 10002 Cáceres, Spain; jjgomezbarrado@gmail.com; 4 Physical Medicine and Rehabilitación Department, San Pedro de Alcántara Hospital, Avda. Pablo Naranjo S/N, 10002 Cáceres, Spain; victormabello@gmail.com; 5 Sports Rehabilitation Area, Sohail Clinic, Avda. Santa Amalia 24, 29640 Fuengirola, Spain; cdiazcasasola0@gmail.com

**Keywords:** acute coronary syndrome, CRP, motivation, HRQoL, tennis

## Abstract

Background: The objective is to analyse and compare the effects of an adapted tennis cardiac rehabilitation programme and a classical bicycle ergometer-based programme on the type of motivation towards sports practice and quality of life in patients classified as low risk after suffering acute coronary syndrome. Methods: The Behavioural Regulation in Exercise Questionnaire (BREQ-2) and Velasco’s Qualityof Life Test were applied. The sample comprised 110 individuals (age = 55.05 ± 9.27) divided into two experimental groups (tennis and bicycle ergometer) and a control group. Results: The intra-group analysis showed a significant increase between pre- and post-test results in intrinsic regulation in the tennis group and in the control group. In identified regulation, the bicycle ergometer group presented significant differences from the control group. On the other hand, in the external regulation variable, only the tennis group showed significant differences, which decreased. Significant improvements in all quality-of-life factors when comparing the pre-test period with the post-test period were only found in the experimental groups. As per the inter-group analysis, significant differences were observed in favour of the tennis group with respect to the control group in the variables of health, social relations and leisure, and work time as well as in favour of the bicycle ergometer group compared with the control group in the variables of health, sleep and rest, future projects and mobility. No significant differences were found in any of the variables between the tennis group and the bicycle ergometer group. Conclusion: It is relevant to enhance the practice of physical exercise in infarcted patients classified as low risk as it improves the forms of more self-determined regulation towards sporting practice and their quality of life.

## 1. Introduction

Cardiac rehabilitation (CR) is a programme supervised by professionals that helps people recover from heart attacks and their associated surgery [1]. It has been shown to be an effective tool for improving body composition [2,3], functional capacity and physical fitness [4,5,6], heart rate variability (HRV) [7,8] and health-related quality of life (HRQoL) [9,10]. However, and despite the unquestionable benefits provided by CRPs, a study performed in 28 European countries concluded that less than half of the cardiovascular patients benefited from a CRP [11], and, in fact, the benefits were generally transitory as patients dropped out of the programmes [12].

On the other hand, several studies have shown the relationship between motivation and the degree of participation in CRPs [13]. Several studies have concluded that if the level of motivation during the CRP is high, then a higher maintenance rate of physical activity, post-CRP, is obtained [14]. Thus, in sport psychology, different approaches have been used to address the analysis of motivational variables, giving considerable importance to the perspective of the Self-Determination Theory (SDT) [15,16]. Our study is based on this theoretical framework.

According to SDT, it is crucial to understand the different forms of intrinsic motivation and the contextual factors that either promote or prevent the internalisation and integration of behaviours. This process depicts a continuum ranging from amotivation (lack of intention to act) to extrinsic motivation (behaviours regulated by external agents) and intrinsic motivation (prototypal self-determination behaviour). Different studies conducted in the physical and sports domain have shown that meeting these three needs increases self-determined motivation, which can be identified as the enjoyment in the activity (intrinsic regulation), and the evaluation and recognition of its importance (identified regulation) [17].

As per the cardioprotective character of tennis, research has shown that racquet sports participation is associated with a significantly reduced risk of CVD (cardiovascular disease) mortality of 56% [18]. On the other hand, when looking at the associations between vigorous and moderate intensity physical activity and the risk of major chronic disease among 44,551 men aged 40–75 years during 22 years of follow-up, it has been shown that playing tennis is associated with a lower risk of major chronic disease and CVD [19].

Therefore, it could be interesting to consider a CRP involving sports that complement the available choice of traditional cardiac rehabilitation programmes (ergometer bicycles, gymnastic tables) to suit different motivations and interests and to increase the patients’ participation in CRPs and their adherence to physical exercise [2].

Therefore, the main objective of this study is to analyse and compare the effects of an adapted tennis CRP and those of a classical bicycle ergometer-based CRP on the type of motivation towards sporting practice and the HRQoL of low-risk patients who have suffered acute coronary syndrome (ACS).

The first hypothesis raised was that “patients with ACS who took part in CRPs using an adapted tennis programme based on trying to maintain constant intensity as well as a classical bicycle ergometer CRP would show a greater increase in HRQoL than those who did not take part in such programme”.

The second hypothesis was that “the participation of patients with ACS in an adapted tennis CRP would increase the levels of more self-determined regulation more than those taking part in a classical bicycle ergometer CRP”.

The third hypothesis was that “patients with ACS who participated in CRPs, either using the adapted tennis programme or the bicycle ergometer programme, would show a greater increase in terms of more self-determined regulation and HRQoL than patients who did not engage in guided physical exercise”.

## 2. Materials and Methods

### 2.1. Participants

The sample comprised 110 patients with ages ranging between 29 and 75, with an average age of 55.05 ± 9.27, 100 of whom were men (age = 54.70 ± 9.10) and 10 women (age = 58.60 ± 10.65). They were distributed into the tennis group (*n* = 45), the bicycle ergometer group (*n* = 35), and the control group (*n* = 30). The baseline characteristics of the patients of the three groups are presented in Table 1.

All patients had ACS and were treated at the coronary unit of a public hospital and classified as low risk according to the criteria of the American Association of Cardiovascular and Pulmonary Rehabilitation [20]. This association classifies this risk into three levels (low, intermediate, and high) and defines the main characteristics of low-risk coronary patients as follows: hospital course without complications, no ischemia or severe ventricular arrhythmias, functional capacity >7 metabolic equivalents (METS) and ejection fraction (EF) >50%. The main inclusion criteria for the study were: belonging to the health area, being over 18 years of age, having suffered an acute myocardial infarction (AMI), not having any other pathology that would prevent carrying out the CRP, and signing the informed consent. The exclusion criteria were: suffering from any chronic disease or organic impairment, suffering from an invalidating psychiatric disorder, having undergone major surgery in the previous six months, and not signing the informed consent.

### 2.2. Measures

The version of the Behavioural Regulation in Exercise Questionnaire-2 (BREQ-2) [21], translated into Spanish [22], was used to measure the levels of self-determined motivation. This instrument has 19 items and 5 factors that represent different types of motivation. Starting with the highest degree of self-determined regulation, the types of motivation are classified as follows: intrinsic regulation (four items: e.g., “I do physical exercise because I think that exercise is fun”); identified regulation (four items: e.g., “I do physical exercise because I value the benefits of doing exercise”); introjected regulation (three items: e.g., “I do physical exercise because I feel guilty if I don’t do it”); external regulation (four items: e.g., “I do physical exercise because other people tell me that I have to do it”), and amotivation (four items: e.g., “I don’t see why I have to do it “). All the items were answered using a Likert type scale, ranging from a value of 0 (not at all true) to 5 (totally true).

Likewise, to determine HRQoL in post-infarction patients, a questionnaire was used [23]. This questionnaire was based on Oldridge’s specific QLMI and on two generic questionnaires (Sickness Impact Profile (SIP) and Quality of Well-Being Questionnaire by Kaplan); it was adapted to the cultural context and had been used with patients after acute myocardial infarction (AMI) in different studies [24,25]. It is designed to be self-administered, and it is comprised of 44 items, with 9 scales: perceived health, sleep and rest, emotional behaviour, future projects, mobility, social relations, alert behaviour, communication, and leisure and work time. All the items were answered using a Likert type scale, with values ranging from 1 (never present) to 5 (always present). Based on the above, the higher the score, the lower the HRQoL, and vice versa.

The approximate time used to complete the questions was about 20 min.

### 2.3. Procedure

**Figure 1:**
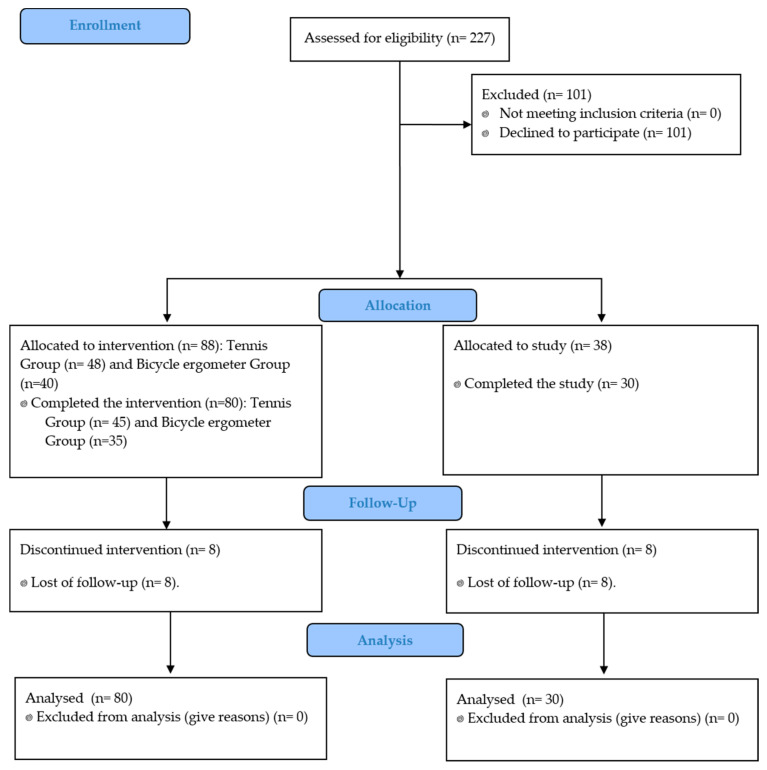
Flow chart of participants.

Figure 1 shows the flow diagram of the participants. Participation in the CRP was offered to all patients (total: 227) in a meeting to inform them of the conditions and objectives of the study. A total of 126 decided to participate in the study (55.51%), and a total of 101 (44.49%) did not want to participate in the intervention group or in the control group. The percentage of participants that completed the full data collection was 87.30% (110/126). The patients who decided not to take part had different reasons (e.g., going back to work soon; living a long way from the programme venue—it is the only CRP in a region with a large geographical extension; no desire to carry it out but were willing to carry out the initial and final tests were deemed part of the control group). In total, 88 individuals expressed interest in participating in the CRP and 38 in the control group (total: 126). The type of sampling used in this study was intentional. Consequently, the 88 patients who were willing to be part of one of the two intervention groups (tennis or bicycle ergometer) were blindly randomised in a proportion of 6 and 5 individuals for each sub-group of tennis and bicycle ergometer, respectively, considering methodological reasons in the case of tennis and the limitation of five bicycle ergometers in the hospital. Hence, 8 sub-groups of 5 patients were established for the bicycle ergometer group (40) and 8 sub-groups of 6 patients for the tennis group (48). The same guidelines were followed as in other studies on patients with very similar characteristics to those of our CRP, which is offered to all patients because of the benefits of CRPs [25]. The patients who agreed to participate signed the informed consent, and the study was approved by the bioethics committees of the hospital and the university, according to the guidelines of the Declaration of Helsinki (World Medical Association).

Moreover, a quasi-experimental design was used with the two experimental groups and the control group [26].

The 3 groups had the following characteristics:

(a) First experimental group: development of a CRP, with a maximum of 8 patients per group, with tennis being the main physical activity (indoor court).
(b) Second experimental group: development of a CRP, with a maximum of 8 patients per group, with the bicycle ergometer as the main activity. This was carried out at the hospital cardiac rehabilitation unit.
(c) Control group: the patients in this group were informed of certain general recommendations, but they did not receive any guided or supervised physical training, as did the other two groups.

The cardiac rehabilitation programme consisted of four main phases:

PHASE 1: During their stay in hospital (3–5 days), the cardiologist carried out the medical history and explained what the CRP consisted of. A complete study of the patients’ state of health was performed, including their evolution and recovery and evaluating possible treatment options (surgical, pharmacological).

PHASE 2: Two weeks later, patients performed a stress test and were later referred to the rehabilitation service to check that their neuronal and muscle-skeletal activity was adequate, proceeding with the initial tests.

PHASE 3: Patients were trained with the bicycle ergometer for one month at the hospital rehabilitation unit, where they were supervised individually by a rehabilitation doctor and a physiotherapist. The aim of this phase was to reduce the risk of muscle–skeletal problems and improve the individuals’ general physical state.

PHASE 4: In this phase, which lasted two months, patients from the two experimental groups began to carry out the training protocol of their group. Three weekly 60-min training sessions (28 in all) were held for each activity. The bicycle ergometer training was carried out at the hospital rehabilitation unit under the supervision of the rehabilitation doctor and the physiotherapist of the research team, whilst the tennis training took place on an indoor tennis court, under the supervision of sports science graduates, a cardiologist and a rehabilitation doctor. All the sports science graduates had previously participated in a 63-h official university advanced course on the development of a CRP via adapted tennis. They had also done a course on handling automated external defibrillators (AED) and on cardiopulmonary resuscitation (CPR). Finally, they took part in a training phase prior to carrying out the CRP. In both groups, the exercise intensity depended on the results obtained in the previous ergometry, around 70–85% of HRmax, trying to maintain the patients as long as possible in that working zone. This was controlled using Polar S610I heart rate monitors. Each session was comprised of a 10-min warm-up, 25–35 min of training, 5 min for the heart rate to drop and 10 min of dynamic stretching of the upper and lower muscle groups. The exercises, especially those with greater technical difficulty, were modified according to the individual evolution of the patients’ skill level. During the tennis lessons, some modifications were included; for example, subjects were allowed to hit the ball after the second bounce at the beginning and mainly playing doubles games rather than singles games. In this context, four different on-court movement intensities were used (according to the patient’s capacity) to maintain the effort within the limits of healthy cardiac output (i.e., walking slowly, walking fast, jogging and running). In this way, the patients would keep moving, following the protocol established by a previous study that converted an intermittent activity such as tennis into a continuous one by adapting its intensity to the areas that had shown significant differences in heart rate (HR) and within areas considered as heart-healthy [2]. This protocol had been successfully tested in a CRP that employed tennis training sessions and rehabilitation instruments for patients after acute myocardial infarction [27].

The aforementioned questionnaires were handed out at the same time to all patients of the sub-groups: at the beginning and at the end of the programme. This was done by two assessors who had experience in administering questionnaires and had received prior training so that, among other issues, they could answer possible doubts from the patients. Patients completed the questionnaires anonymously and confidentially. The approximate time used to answering the questionnaires was about 20 min.

### 2.4. Statistical Analysis

Statistical analysis was conducted using the SPSS statistical package (Statistical Package for Social Sciences, version 25 for Windows, IBM Corporation, Armonk, NY, USA).

Factor analysis was performed to determine the validity of the different instruments. Reliability analyses were performed to create the different variables. The Kolmogorov–Smirnov’s test was used to check normality; homogeneity of variance was checked using Levene’s test, and a normal distribution of data was observed.

In the pre-test measurement, it was determined that there were no significant differences in the majority of the variables between the selected groups.

To establish the statistical differences between groups, a repeated-measure ANOVA test was used with two factors: one inter-individual factor with three levels (control group, tennis group, bicycle ergometer group) and one intra-individual factor with two levels. Bonferroni’s post-hoc statistical test was performed on those variables that offered significant values in the previous test (*p*-value <0.05) to make a pair-wise assessment of the groups, identifying where the differences between the two lay.

It is important to remember that in the HRQoL variable results, the higher the score, the lower the quality.

## 3. Results

The data from the descriptive and reliability analysis of the variables measured in all individuals participating in the study, both pre-test and post-test, are presented in Table S1 (included as supplementary Materials). Regarding Cronbach’s alpha values, all the factors, with the exception of amotivation in the post-test (0.64), were found to be above the value recommended by Nunnally [28], estimated at 0.70. This value is acceptable in view of the reduced number of items that make up the factor [29]. Skewness kurtosis and scores were acceptable.

On the other hand, the descriptive statistics regarding motivation showed high levels of the more self-determined regulation factors, with higher means in the post-test. As per the HRQoL factors, the lowest factors were found in the post-test, meaning that patients with ACS displayed better HRQoL at the end of the study.

In addition to the pre- and post-test differences between the groups, the inter-group differences in motivation and HRQoL are presented in Table 2. The magnitude of the effect size (η^2^) is small in all variables, in line with Rosenthal [30], who indicates that d = 0.20 is small, d = 0.50 is moderate, and d = 0.80 is large.

In terms of motivation, in the intra-group comparison, the tennis group presented significant differences pre-and post-CRP in the variables of intrinsic regulation, which increased (*p* < 0.01), and external regulation, which decreased (*p* < 0.02). The bicycle ergometer group presented significant differences in the variable of identified regulation, which increased (*p* < 0.03). On the other hand, the control group presented significant differences in intrinsic regulation, which increased (*p* < 0.01), and identified regulation, which increased (*p* < 0.01).

With respect to HRQoL, in the intra-group comparison, the results of the experimental groups of tennis and bicycle ergometer improved with respect to all variables after the CRP. On the other hand, the control group improved with respect to all variables but only significantly in the variables of perceived health, future projects, mobility, and social relations. As per the inter-group contrast, significant differences were observed in the variables of health (*p* < 0.01), sleep and rest (*p* < 0.05), future projects (*p* < 0.03), mobility (*p* < 0.01), social relations (*p* < 0.01), and leisure and work time (*p* < 0.01).

Table 3 shows the results of the pair-wise post-hoc analysis of the groups regarding those variables that offered significant values in the previous analysis (*p*-value < 0.05); all of them related to HRQoL. Significant differences were observed in favour of the tennis group with respect to the control group in the variables of health (*p* < 0.03), social relations (*p* < 0.01) and leisure and work time (*p* < 0.01). On the other hand, significant differences were observed in favour of the bicycle ergometer group compared with the control group in the variables of health (*p* < 0.03), sleep and rest (*p* < 0.05), future projects (*p* < 0.05) and mobility (*p* < 0.05). Finally, no significant differences were found in any of the variables between the tennis group and the bicycle ergometer group.

## 4. Discussion

This study analyses the changes that occur in the terms of self-determined regulation and in HRQoL of patients with ACS after carrying out guided physical exercise, either through an adapted tennis-based CRP or a classical bicycle ergometer-based CRP. The results will be discussed according to the hypotheses formulated.

The first hypothesis, which indicated that “patients with ACS who participate in CRPs using adapted tennis based on trying to maintain a constant intensity as well as a classical bicycle ergometer CRP would show a greater increase in HRQoL”, was met since in all HRQoL factors comparing the pre-test period with the post-test period were only found in the experimental groups; no significant differences were found in any of the variables between the tennis group and the bicycle ergometer group. These results seem especially interesting since the results of a previous study showed that the physiological demands of advanced and recreational veteran men’s tennis players during an hour of tennis match play, independently of ability, satisfied the need for quantity and quality of exercise for the development and maintenance of cardiovascular fitness in healthy adults [31]. Furthermore, another study showed that elderly players who had practised the sport for a considerable time exhibited relatively lower arterial stiffness and lower insulin resistance compared to those with lower time tennis-playing [32]. A great concern was whether the actual practice time during the development of the tennis sessions, a discontinuous activity, could be close to that of continuous activity, such as the cycle ergometer, to cause similar benefits in the HRQo. Thus, the protocol that maintains the effort within the limits of healthy cardiac output (i.e., walking slowly, walking fast, jogging and running) [2] appears to be effective.

The second hypothesis, which stated that “the participation of patients with ACS in an adapted tennis CRP would increase the levels of more self-determined regulation more than a classical bicycle ergometer CRP”, was met. The intra-group analysis showed a significant increase between pre- and post-test values in intrinsic regulation in the tennis group and an increase in the identified regulation in the bicycle ergometer group. It should be emphasised that the only group where the external regulation factor decreased significantly was the tennis group, with similar results to those who carried out physical activity without any type of obligation [33,34]. Therefore, studies have concluded that an increase in self-directed motivation may enhance the motivation for physical activity, leading to increased adherence to physical activity and healthier lifestyles [35]. In other words, patients from this tennis group did not carry out physical activity because they had suffered a stroke and because there was an (external) medical prescription that indicated that exercise was essential for their recovery or to prevent the appearance of more strokes. In this regard, the CRPs carried out with guided physical exercise are effective in favouring changes towards healthy and long-lasting habits in stroke patients [5]. The fact that the tennis group presents better results than the cycle ergometer group may be due to the interest that doing an activity such as tennis outside the hospital, playing with other patients, playing for points and so forth can generate. This would complement the available choice of traditional programs of cardiac rehabilitation (ergometer bicycles, treadmills, gymnastic tables) to suit different motivations and interests and increase the participation of patients in CRPs and the adherence to physical exercise [2]. The control group significantly increased the more self-determined regulations, probably because they carried out physical activity individually, as prescribed by the physician.

The third hypothesis, which was that “patients with ACS who participate in CRPs, either using the adapted tennis programme or the bicycle ergometer programme, would show a greater increase in the form of more self-determined regulation and HRQoL than patients who do not perform guided physical exercise”, was partially met. Indeed, no significant differences were found in the more self-determined forms of regulation between either of the two experimental groups with respect to the control group. There were significant differences in some of the HRQoL factors (health, social relations, leisure and work time) of the experimental group that carried out a tennis CRP with respect to the control group and the experimental group that carried out a classical CRP with respect to the control group in the HRQoL factors of health, sleep and rest, future projects and mobility.

In line with the second hypothesis, the lack of significant differences with respect to the more self-determined forms of regulation between the groups was due to the fact that the control group could also carry out physical activity on its own, following the medical advice of our research team. Therefore, future studies should analyse and control the physical activity of this group. The data obtained in this research related to HRQoL, coinciding with the findings of other studies [9,25]. The latter study used the same measurement instrument as ours on, similarly, patients who had suffered ACS and were classified as low risk. In this case, the patients who participated in the CRP showed significant improvements in all the factors of HRQoL.

Finally, as per the limitations of the study, it should be noted that the low participation of women in our CRP is in line with other studies, such as that of Espinosa et al. [25], with 153 low-risk patients with myocardial infarct who participated in a CRP, of whom only 10 were women. Thus, apart from the fact that the probability of suffering a stroke is double in women than in men, this considerable difference in participation may be due to the fact that women have to overcome specific challenges to participate in the CRP, including playing the role of informal caregivers (children, grandchildren, a relative dependent on them in the household), being on-demand for multiple roles, having a negative body image, lack of previous experience with exercise, and limited cultural support for physically active lifestyles [24].

Another limitation found was the difficulty for patients to travel from the different points of the region to carry out the program, as it is the only existing CRP in the region. Hence, very few women were included in the program due to the reasons mentioned above. Furthermore, another limitation of the study was the possibility that improvements in HRQoL may have been due to time that had elapsed since the patients suffered acute coronary syndrome, as can be evidenced by improvements in the control group. Another drawback of the study was that patients were not randomly assigned to the control group, which consequently affected the homogeneity of the groups. This was because the CRP was offered to all patients due to the aforementioned important benefits to the cardiovascular health of those participating in these programs. In our study, the control group was determined by patients who did not want to participate in a CRP but who were willing to participate in initial and final evaluations. However, possible differences between groups were verified for baseline values. No differences were found in the age of the participants, and, of the fourteen factors analysed in our study (five for motivation and nine for HRQoL), differences were only found in the intrinsic regulation factor; no differences between groups in this factor were found post-test.

Another important aspect to be considered in the development of CRPs by adapting tennis or other sports is its possible extra cost compared to traditional cardiac rehabilitation programs (e.g., ergometer bicycles, treadmills, gymnastic tables). Developing innovative CRPs by adapting sports could favour sustainability strategies that include the use of public sports facilities that are often under-used and the implementation of programs in nature. In the case of this study, the tennis facility and equipment were loaned by the university, and the coaches’ fees were not higher than those of other health professionals.

Future perspectives of this study could consider the inclusion of an extinction measurement three months after the intervention program and even a long-term follow-up to better understand the extent to which the patients have carried on with these habits on their own, without continuing within the CRP. Furthermore, other study variables could be introduced, such as the measurement of the participants’ anxiety, self-esteem and physical fitness. Finally, the development of one single CRP combining different individual or team sports (swimming, dancing, basketball) could well be accepted by a larger number of patients, especially women, providing greater benefits to motivation and HRQoL levels.

## 5. Conclusions

CRPs of either adapted tennis or classical exercise are very useful to increase the more self-determined forms of motivation and favour an improvement in the HRQoL of patients with ACS. Therefore, we recommend the implementation of alternative programs to the classical ones that use bicycle ergometers, such as the adapted tennis program presented in this research or other sports adapted to the participants’ interests.

## Figures and Tables

**Table 1:** Baseline characteristics of the patients.

Participants	Tennis Group	Bicycle Ergometer Group	Control Group
Sample Size and Age			
Sample size (N)	45	35	30
Age (years)	56.62 ± 9.29	53.06 ± 9.11	55.03 ± 9.28
ST-segment elevation acute coronary syndrome (NSTE-ACS)			
Non STE-ACS	21/45 (46.7%)	8/45 (17.8%)	16/45 (35.5%)
Anterior STE-ACS	18/45 (40%)	11/45 (24.4%)	16/45 (35.6%)
Inferior STE-ACS	19/45 (42.2%)	12/45 (26.7%)	14/45 (31.1%)
Risk factors for cardiovascular disease			
Smoker	37 (82.2%)	26 (74.3%)	25 (83.3)
Arterial hypertension	18 (40%)	13 (37.1%)	13 (43.3%)
Obesity	35 (77.8%)	26 (74.3%)	25 (83.3%)
Dyslipidemia	34 (75.5%)	28 (80%)	26 (86.7%)
Sedentary lifestyle	41 (91.1%)	30 (85.7%)	27 (90%)
Diabetes mellitus	9/45 (20%)	6/35 (17.1%)	7/30 (23.3%)
Medication			
Beta blockers	41 (91.1%)	32 (91,4%)	28 (93.3)
Statins	43 (95.5%)	33 (94.3%)	29 (96.7%)

**Table 2:** Differences inter-groups and intra-groups in HRQoL and motivation.

	Tennis Group			Bicycle Ergometer Group			Control Group			Contrast Inter-Groups	
Variables		M ± SD	*P*		M ± SD	*P*		M ± SD	*P*	F	η^2^
**Motivation**											
Intrinsic Regulation	Pre	3.99 ± 0.85	0.00	Pre	3.96 ± 0.83	0.08	Pre	3.26 ± 0.17	0.00	36	0.00
	Post	4.60 ± 0.53		Post	4.46 ± 0.46		Post	3.72 ± 0.18			
Identified Regulation	Pre	4.53 ± 0.79	0.43	Pre	4.53 ± 0.68	0.03	Pre	3.87 ± 0.13	0.00	1.11	0.02
	Post	4.64 ± 0.63		Post	4.82 ± 0.31		Post	4.23 ± 0.16			
Introjected Regulation	Pre	3.18 ± 1.42	0.47	Pre	3.26 ± 1.35	0.64	Pre	2.9 ± 0.18	0.65	1.15	0.02
	Post	3.01 ± 1.39		Post	3.60 ± 1.28		Post	2.84 ± 0.26			
External Regulation	Pre	2.11 ± 1.17	0.02	Pre	1.96 ± 0.98	0.38	Pre	2.59 ± 0.18	0.06	0.83	0.01
	Post	1.71 ± 0.87		Post	1.89 ± 1.22		Post	2.19 ± 0.20			
Amotivation	Pre	1.72 ± 0.96	0.13	Pre	1.55 ± 0.78	0.75	Pre	1.67 ± 0.11	0.10	2.42	0.04
	Post	1.47 ± 0.60		Post	1.44 ± 0.67		Post	1.93 ± 0.17			
HRQoL											
Perceived health	Pre	2.25 ±.76	0.00	Pre	2.35 ± 0.11	0.00	Pre	2.12 ± 0.13	0.01	9.03	0.14
	Post	1.61± 0.47		Post	1.67 ± 0.08		Post	1.95 ± 0.12			
Sleep and rest	Pre	2.17 ± 1.12	0.00	Pre	2.29 ± 0.12	0.00	Pre	2.02 ± 0.16	0.11	3.24	0.05
	Post	1.68 ± 0.76		Post	1.60 ± 0.08		Post	1.88 ± 0.14			
Emotional behaviour	Pre	2.13 ± 1.04	0.00	Pre	1.96 ± 0.14	0.00	Pre	2.43 ± 0.21	0.39	2.90	0.05
	Post	1.54 ± 0.70		Post	1.55 ± 0.11		Post	2.32 ± 0.19			
Future projects	Pre	2.31 ± 0.97	0.00	Pre	2.43 ± 0.19	0.00	Pre	2.10 ± 0.18	0.00	4.76	0.08
	Post	1.56 ± 0.60		Post	1.51 ± 0.13		Post	1.78 ± 0.12			
Mobility	Pre	2.17 ± 1.06	0.00	Pre	2.10 ± 0.11	0.01	Pre	1.97 ± 0.14	0.00	7.82	0.00
	Post	1.46 ± 0.56		Post	1.24 ± 0.05		Post	1.78 ± 0.13			
Social relations	Pre	2.06 ± 0.79	0.00	Pre	1.88 ± 0.10	0.01	Pre	2.06 ± 0.16	0.00	4.88	0.08
	Post	1.44 ± 0.50		Post	1.38 ± 0.07		Post	1.85 ± 0.14			
Alert behaviour	Pre	2.21 ± 1.13	0.00	Pre	1.91 ± 0.15	0.00	Pre	1.88 ± 0.17	0.08	1.96	0.035
	Post	1.76 ± 0.81		Post	1.54 ± 0.13		Post	1.74 ± 0.14			
Communication	Pre	1.72 ± 0.94	0.00	Pre	1.66 ± 0.15	0.00	Pre	1.88 ± 0.18	0.09	1.53	0.028
	Post	1.36 ± 0.70		Post	1.21 ± 0.10		Post	1.73 ± 0.16			
Leisure and work time	Pre	2.47 ± 0.97	0.00	Pre	2.35 ± 0.16	0.00	Pre	2.21 ± 0.18	0.25	9.06	0.145
	Post	1.64 ± 0.63		Post	1.73 ± 0.11		Post	2.11 ± 0.14			

M ± SD: Mean ± Standard Deviation. *P*: Significant differences intra-group. Pre: before the program. Post: after the program. F: Fisher’s F-distribution. η^2^: Measure of effect size Eta-squared.

**Table 3:** Pair-wise post-hoc analysis.

Pair-Wise Comparisons		Perceived Health	Sleep and Rest	Future Projects	Mobility	Social Relations	Leisure and Work Time
Tennis Group	Control Group	−0.48 ± 0.13 **	−0.34 ± 0.20	−0.43 ± 0.19	−0.52 ± 0.17	−0.41 ± 0.13 ***	−0.74 ± 0.17 ***
Bicycle ergometer Group	Tennis Group	−0.36 ± 0.12	−0.20 ± 0.19	−0.17 ± 0.18	−0.16 ± 0.16	0.11 ± 0.13	0.20 ± 0.17
	Control Group	−0.51± 1.35 **	−0.54 ± 0.21 *	−0.59 ± 0.20 *	−0.68 ± 0.18 *	−0.30 ± 0.14	−0.53 ± 0.18

* *p*-value <0.05; ** *p*-value <0.03; *** *p*-value <0.01.

## Data Availability

Data will be available upon reasonable request to corresponding author.

## Supplementary Materials

The following are available online at https://www.mdpi.com/article/10.3390/ijerph18179207/s1, Table S1: Descriptive Statistics and Reliability Analysis.
