# Supplementary material for: Modification of the Forms of Self-Determined Regulation and Quality of Life after a Cardiac Rehabilitation Programme: Tennis-Based vs. Bicycle Ergometer-Based
Source: Int J Environ Res Public Health. 2021 Aug 31;18(17):9207. doi: 10.3390/ijerph18179207 (PMC8430765; doi:10.3390/ijerph18179207)
Supplement: Supplementary file 1 [file ijerph-18-09207-s001.zip › ijerph-1344917-supplementary.pdf]

Table S1. Descriptive Statistics and Reliability Analysis.

Variables	M ± SD	α	Skewness	Kurtosis
BREQ-2 PRE				
Intrinsic	3,78 ± 0.92	0.81	-0.64	-0.27
Identified	4.35± 0.79	0.82	-1.14	0.44
Introjected	3.14 ± 1.29	0.79	-0.12	-1.01
External	2.19 ± 1.08	0.76	0.27	-1.22
Amotivation	1.65 ± 0.81	0.75	1.14	0.39
BREQ-2 POST				
Intrinsic	4.32 ± 0.75	0.84	-1.48	2.23
Identified	4.58± 0.68	0.74	-2.58	8.80
Introjected	3.15 ± 1.38	0.78	-0.34	-1.06
External	1.89 ± 1.06	0.82	0.95	-0.40
Amotivation	1.58 ± 0.74	0.64	1.29	0.87
Q. LIFE PRE				
Perceived health	2.25 ± 0.70	0.82	0.59	-0.09
Sleep and rest	2.17 ± 0.94	0.87	0.62	-0.04
Emotional behaviour	2.16 ± 1.02	0.89	0.41	-0.95
Future projects	2.29 ± 1.02	0.76	0.34	-0.92
Movility	2.10 ± 0.88	0.90	0.84	0.47
Social relations	2.00 ± 0.77	0.84	0.66	-0.45
Alert behaviour	2.02 ± 1.01	0.89	1.24	1.14
Communication	1.75 ± 0.94	0.95	1.71	2.83
Leisure and work time	2.36 ± 0.97	0.90	0.30	-0.70
Q. LIFE POST				
Perceived health	1.72 ± 0.53	0.83	1.14	1.24
Sleep and rest	1.70 ± 0.68	0.75	0.95	0.44
Emotional behaviour	1.75 ± 0.85	0.86	0.91	-0.44
Future projects	1.60 ± 0.73	0.78	1.44	1.56
Movility	1.47 ± 0.57	0.89	1.54	2.53
Social relations	1.53 ± 0.59	0.86	1.53	1.75
Alert behaviour	1.68 ± 0.78	0.82	1.41	1.73
Communication	1.41 ± 0.74	0.95	2.41	6.20
Leisure and work time	1.79 ± 0.70	0.85	0.83	0.09
